# Activation of Relaxin Family Receptor 1 from Different Mammalian Species by Relaxin Peptide and Small-Molecule Agonist ML290

**DOI:** 10.3389/fendo.2015.00128

**Published:** 2015-08-17

**Authors:** Zaohua Huang, Courtney Myhr, Ross A. D. Bathgate, Brian A. Ho, Amaya Bueno, Xin Hu, Jingbo Xiao, Noel Southall, Elena Barnaeva, Irina U. Agoulnik, Juan J. Marugan, Marc Ferrer, Alexander I. Agoulnik

**Affiliations:** ^1^Department of Human and Molecular Genetics, Herbert Wertheim College of Medicine, Florida International University, Miami, FL, USA; ^2^Department of Biochemistry and Molecular Biology, Florey Department of Neuroscience and Mental Health, Florey Institute of Neuroscience and Mental Health, The University of Melbourne, Melbourne, VIC, Australia; ^3^NIH Chemical Genomics Center, National Center for Advancing Translational Sciences, National Institutes of Health, Rockville, MD, USA; ^4^Department of Cellular Biology and Pharmacology, Herbert Wertheim College of Medicine, Florida International University, Miami, FL, USA

**Keywords:** relaxin, G protein-coupled receptor, RXFP1, receptor structure–function, small-molecule allosteric agonist

## Abstract

Relaxin peptide (RLN), which signals through the relaxin family peptide 1 (RXFP1) GPCR receptor, has shown therapeutic effects in an acute heart failure clinical trial. We have identified a small-molecule agonist of human RXFP1, ML290; however, it does not activate the mouse receptor. To find a suitable animal model for ML290 testing and to gain mechanistic insights into the interaction of various ligands with RXFP1, we have cloned rhesus macaque, pig, rabbit, and guinea pig RXFP1s and analyzed their activation by RLN and ML290. HEK293T cells expressing macaque or pig RXFP1 responded to relaxin and ML290 treatment as measured by an increase of cAMP production. Guinea pig RXFP1 responded to relaxin but had very low response to ML290 treatment only at highest concentrations used. The rabbit RXFP1 amino acid sequence was the most divergent, with a number of unique substitutions within the ectodomain and the seven-transmembrane domain (7TM). Two splice variants of rabbit RXFP1 derived through alternative splicing of the fourth exon were identified. In contrast to the other species, rabbit RXFP1s were activated by ML290, but not with human, pig, mouse, or rabbit RLNs. Using FLAG-tagged constructs, we have shown that both rabbit RXFP1 variants are expressed on the cell surface. No binding of human Eu-labeled RLN to rabbit RXFP1 was detected, suggesting that in this species, RXFP1 might be non-functional. We used chimeric rabbit–human and guinea pig–human constructs to identify regions important for RLN or ML290 receptor activation. Chimeras with the human ectodomain and rabbit 7TM domain were activated by RLN, whereas substitution of part of the guinea pig 7TM domain with the human sequence only partially restored ML290 activation, confirming the allosteric mode of action for the two ligands. Our data demonstrate that macaque and pig models can be used for ML290 testing.

## Introduction

The relaxin hormone was discovered by Dr. Frederick Hisaw 90 years ago in experiments involving the injection of serum from pregnant guinea pigs or rabbits into virgin guinea pigs that resulted in the softening of the pubic ligament (1). Further experiments led to the identification of the peptide responsible for this effect. It was the first peptide hormone identified in mammals. The relaxin gene, RLN1, has a relatively simple structure, containing only two exons (2). The mRNA encodes the preprohormone, which is processed by convertases to the mature 6 kDa hormone with A- and B-chains connected to each other by two disulfide bonds. An additional disulfide bond is located within the A-chain. Relaxin (RLN) peptides from various mammalian species show significant variations in amino acid sequence; however, almost all maintain the conserved functional RXXXRXXI/V motif in the B-chain. The analysis of the full genome sequences revealed the presence of only one RLN gene in various species. One exception is primates, where two RLN1 and RLN2 genes coding for almost identical peptides are located next to each other, most likely the result of genomic DNA duplication. It is believed that the RLN2 gene is the functional copy, as only the RLN2 peptide was isolated from the peripheral blood (1).

The cognate receptor for RLN peptide is the G protein-coupled receptor Relaxin Family Peptide Receptor 1, or RXFP1 (3). All *RXFP1* genes cloned to date from various mammalian species have the same conserved 18-exon genomic organization and encode proteins with very similar structures. RXFP1 contains a large extracellular ectodomain, which is unique among G protein-coupled receptors. This domain consists of a single low-density lipoprotein receptor type A module (LDLa) followed by 10 leucine-rich repeats (LRRs). The classical seven-transmembrane (7TM) region of the RXFP1 is well-conserved among different species. Structural studies of RLN and RXFP1 binding and activation have revealed a complex mechanism of their interaction (2). It was established that primary high-affinity binding of RLN occurs within the LRRs, while the secondary low-affinity interaction occurs via the second extracellular loop (ECL) of the 7TM region. The LDLa domain is not necessary for binding but is essential for activation of the receptor signaling, although the detailed mechanism of these interactions is still under investigation. When transfected into HEK293T, CHO, or other cells, human, mouse, and rat RXFP1s respond to RLN treatment by increasing cAMP production. Increased phosphorylation of extracellular signal-regulated kinase 1/2 (ERK1/2), MAPK, tyrosine kinase(s) and activation of nitric oxide (NO) signaling in various RXFP1-transfected cells and cells endogenously expressing RXFP1were also found (2).

Non-reproductive functions of this hormone/receptor pair were identified through analysis of *Rln1-* and *Rxfp1*-deficient transgenic mice, experiments with RLN injection into rodents, and inactivation of RLN signaling using antibodies or peptide antagonists (1, 2). It was shown that RLN behaves as an antifibrotic, antiapoptotic, vasodilatory, and angiogenic factor. This led to the investigation of the therapeutic potential for RLN in several diseases. The most advanced clinical trial to date tested the use of recombinant RLN as a treatment for acute heart failure. The reported analysis suggests that the treatment is well tolerated by patients, safe, and most importantly, results in a reduced 180-day mortality (4).

As with other peptide-based pharmaceuticals, the use of such drugs in chronic conditions is complicated due to their short half-life and the need for intravenous administration. An additional disadvantage is the cost of recombinant peptide production. To overcome these limitations, we have initiated the search for a small-molecule agonist of RXFP1. High throughput screening of a small-molecule library and the subsequent structure activity campaign resulted in the identification of the first series of RXFP1 agonists with preferred biochemical and *in vivo* pharmacokinetic properties, which supported further therapeutic investigation of RLN biology (5, 6). Surprisingly, these compounds, including lead compound, ML290, did not activate the mouse RXFP1 receptor. Using chimeric human–mouse RXFP1 variants and point mutations, we have established that amino acid differences in the third ECL of 7TM are responsible for such specificity (5). This mouse variant is also present in rat and hamster RXFP1s. An overwhelming majority of the preclinical animal testing for RLN treatment includes rodent models, and thus the inability of small-molecule agonists to activate the mouse receptor hampers preclinical studies. To find suitable *in vitro* and *in vivo* models, we have cloned and tested in a functional cAMP assay RXFP1 receptors from four mammalian species: rhesus macaque (Macaca mullata), pig (*Sus scrofa*), European rabbit (*Oryctolagus cuniculus*), and the guinea pig (*Cavia porcellus*). We also tested various chimeric human constructs that had their extracellular or 7TM parts swapped for corresponding guinea pig and rabbit fragments to establish regions responsible for RLN and ML290 activation. For rabbit RXFP1s, which were non-responsive to RLN, we tested surface expression, RLN binding, and activation by RLN peptides from various species. We have concluded that pig and macaque models are suitable for ML290 testing, whereas rodent RXFP1 genes have to be humanized for preclinical studies.

## Materials and Methods

### Sequence analysis

Genomic sequences of the RXFP1 genes for different species were obtained from the Ensembl database^1^. The full-length human and mouse RXFP1 cDNA were used to identify exons using the Blast2seq program (7) available from the NCBI website^2^. Multiple sequence alignments and evolutionary tree rendering were performed using the MUSCLE algorithm (8) at the EMBL-EBI website^3^.

### Production of RXFP1 expression constructs from various species and human–guinea pig or rabbit chimeric RXFP1s

**Figure 1:**
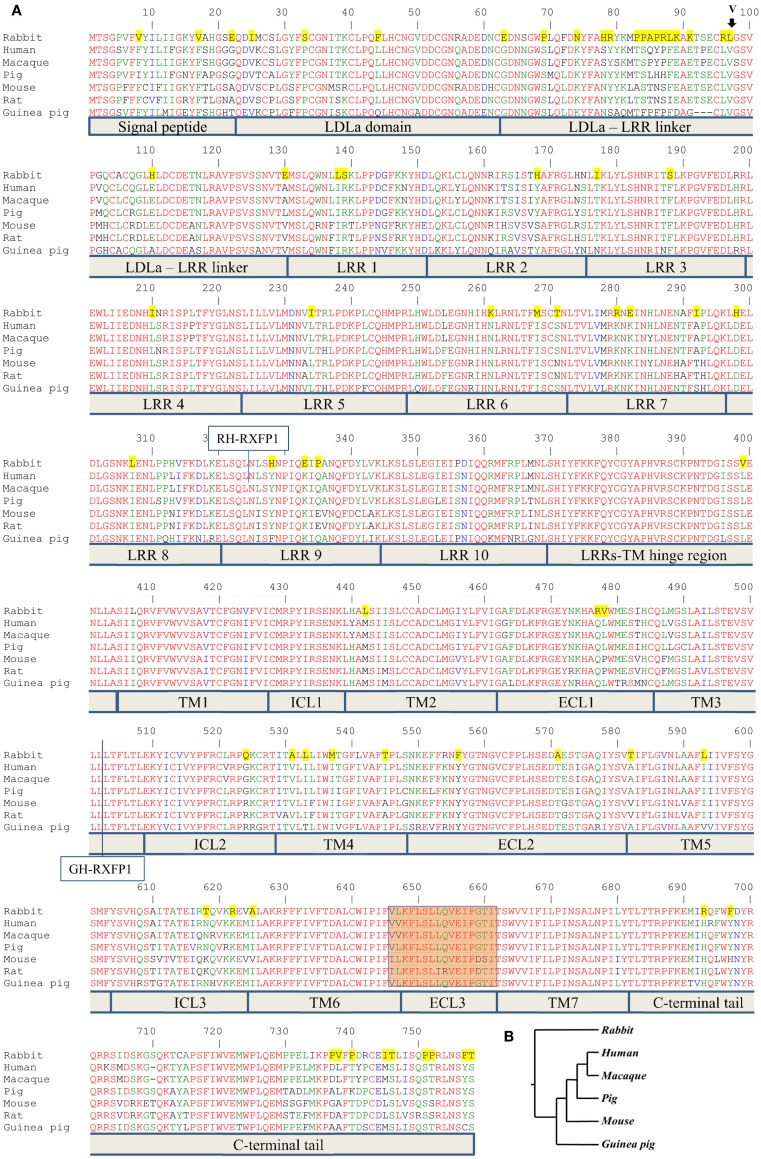
**Alignment of RXFP1 proteins from various species**. **(A)** Amino acid alignment of RXFP1 receptor sequences. The position of the extra amino acid (V98) in rabbit receptor variant R2 is shown above the sequence with an arrow. Functional domains are shown below the sequences. LDLa is low-density lipoprotein class A domain; LRR is leucine-rich repeat, TM1–7 are transmembrane domains; ICL1–3 are intracellular loops of seven-transmembrane domain; ECL1–3 are extracellular loops of seven-transmembrane domain. The highlighted brown box is the third extracellular loop and adjacent amino acids required for ML290 activation of RXFP1. Amino acids conserved in all seven species are in red; amino acids specific for the rabbit sequence are highlighted in yellow. The vertical line at position 324 indicates the fusion site in chimeric rabbit/human receptor (RH-RXFP1). The vertical line at position 502 indicates the fusion site in chimeric guinea pig/human receptor (GH-RXFP1). **(B)** Evolutionary tree showing the relationship of various RXFP1 proteins. The rabbit sequence is the most diverged.

The human, mouse, macaque, and pig RXFP1 cDNA constructs in baculovirus BacMam mammalian expression vector (Invitrogen, Carlsbad, CA, USA) were synthesized at the Eukaryotic Expression Group-Protein Expression laboratory (NCI, Frederick, MD, USA). The PCR primers for RT-PCR were designed to cover the full-length sequence of the open reading frame (ORF). PCR amplifications were performed with *PfuUltra* High-Fidelity DNA polymerase (Agilent Technologies, Santa Clara, CA, USA). Guinea pig *RXFP1* (G-RXFP1) was amplified from guinea pig ovarian cDNA (Zyagen, San Diego, CA, USA). Rabbit *RXFP1* (R1-RXFP1 and R2-RXFP1) cDNAs were amplified from rabbit uterus cDNA (Zyagen). All cloning was performed using the In-Fusion^®^ HD Cloning Kit (Clontech Laboratories, Mountain View, CA, USA). Rabbit and guinea pig *RXFP1*s were cloned into pCR3.1 mammalian expression vector (Invitrogen). In order to study RXFP1 surface expression, rabbit cDNAs were cloned into pcDNA3.1™/Zeo+ mammalian expression vector (Invitrogen), which contained an N-terminal FLAG-tag and a bovine prolactin signal sequence (3). It was shown previously that such additions do not alter receptor activity (14). To make chimeric clones, the human, guinea pig, and rabbit plasmid were used as templates to produce PCR amplicons, which were then used for overlapping PCR and subsequent cloning with the In-Fusion kit. The chimeric guinea pig/human GH-RXFP1 construct contains guinea pig *RXFP1* cDNA (*G-RXFP1*, 1–1499 bp) encoding the ectodomain, TM1, TM2, and part of TM3 (amino acids 1–499 of G-RXFP1), and human RXFP1 (*hRXFP1*, 1509–2274 bp) encoding TM3 to the C-terminal tail of the receptor (amino acids 503–757 of hRXFP1) (Figure 1A). The recombinant chimeric human–rabbit HR-RXFP1 was made with the 5′-part of the *hRXFP1* sequence (1–972 bp, LDLa-LRR9, 1–324 aa), with the remainder being the rabbit *RXFP1* sequence (973–2277 bp, LRR9-C-terminal tail, 325–759 aa) (Figure 1A). The recombinant chimeric rabbit–human R1H-RXFP1 or R2H-RXFP1 contains the N-terminal rabbit RXFP1 sequence (1–972 bp for *R1-RXFP1* or 975 bp for *R2-RXFP1*), with the remaining sequence being *hRXFP1* (973–2274) (Figure 1A). *R1*- and *R2-RXFP1* denote two variants of rabbit *RXFP1* cDNA. All numbers correspond to the full-length cDNAs with the first nucleotide of the ORF.

The rabbit RLN gene SQ10 (9) was synthesized and cloned into pCR2.1-TOPO (Invitrogen) by Eurofins MWG Operon LLC. The SQ10 cDNA was PCR amplified and cloned using In-Fusion kit into a pCR3.1 vector.

At least three independent plasmids were obtained in each cloning experiment. The cDNA inserts were fully sequenced using overlapping primers by Eurofins MWG Operon LLC (Huntsville, AL, USA). GenBank accession numbers are KT149378 (guinea pig RXFP1), KT149379 (rabbit variant 1 RXFP1), and KT149380 (rabbit variant 2 RXFP1).

### Relaxin peptides and cell lines

Porcine relaxin peptide (10) was a gift from Dr. O. David Sherwood (University of Illinois at Urbana-Champaign). Human recombinant RLN peptide was obtained from PeproTech Inc. (Rocky Hill, NJ, USA) or from Corthera (San Carlos, CA, USA). Chemically synthesized mouse RLN peptide was a gift from Prof. John D. Wade (Florey Institute of Neuroscience and Mental Health, Melbourne, VIC, Australia). Human embryonic kidney HEK293T cells (ATCC #CRL-1573; American Type Tissue Culture Collection, Manassas, VA, USA) used for transfection experiments were maintained in 37°C, 5% CO_2_ in Dulbecco’s Modified Eagle medium (DMEM) supplemented with 10% fetal bovine serum (FBS), 1% l-glutamine, and 1% penicillin/streptomycin.

### CRE-Luc BacMam luciferase assay

GloResponse™ CRE-*luc2P* HEK293T cells stably transfected with cAMP response element-driven luciferase (CRE-Luc) reporter (Promega, Madison, WI, USA) were transduced with human, macaque, pig, and mouse RXFP1 expression BacMam vectors according to the manufacturer’s protocol (Invitrogen) and incubated at room temperature for 2 h in the dark with occasional mixing. The volume of the cell culture was adjusted to 1000 cells/μl with DMEM + 10% FBS. Cells (3 μl, 3000 cells) were plated on 1536-well white solid-bottom TC plates and incubated overnight at 37C°, 5% CO_2_. Four hundred micromoles Ro 20-1724 (Sigma-Aldrich, St. Louis, MO, USA) in PBS (1 μl) was added in each well. The cells were treated with serial dilutions of either Forskolin (Sigma-Aldrich), ML290 (5) or porcine RLN at final concentrations’ range (57 μM–3.5 pM) for Forskolin and ML290, and (8.7 μM–1.8 pM, or 57 ng/μl–0.012 pg/μl] for RLN. After 2 h stimulation at 37°C, 4 μl of detection reagent from Amplite™ Luciferase reporter gene assay kit (AAT Bioquest, Sunnyvale, CA, USA) was added as a mix of 90% Component A, 5% Component C, and 5% Component D, and incubated at room temperature for 5 min. Luminescent signal was measured on Viewlux uHTS Microplate Imager (PerkinElmer, Santa Clara, CA, USA). The data were processed using GraphPad Software (San Diego, CA, USA).

### Cell transfection and cAMP assays

HEK293T cell transient transfections were performed using Lipofectamine 2000 transfection reagent (Life Technologies, Grand Island, NY, USA) according to the manufacturer’s instructions. Cells transiently expressing the RXFP1 receptors were used within 48 h of transfection. Each construct was tested at least three times. For cell-conditioned media stimulation, HEK293T cells were transfected with the rabbit RLN construct (SQ10) or an empty pCR3.1 vector, and cells were cultured for 24 h. The media was used to stimulate HEK293T cells transfected with the RXFP1 receptors.

Direct measurement of cAMP production was performed using the HTRF cAMP HiRange kit (CisBio, Bedford, MA, USA). HEK293T cells transiently transfected with different RXFP1 receptors were stimulated with various concentrations of RLN peptides or ML290 for 60 min at 37°C, 5% CO_2_, after which, two HTRF detection reagents (diluted according to assay kit directions in HTRF lysis buffer) were added. The plates were incubated for 60 min at room temperature, and the signal was read on a FLUOstar Omega (BMG Labtech, Cary, NC, USA) plate reader. cAMP levels were calculated according to the manufacturer’s instructions against a standard curve. Statistical processing of the data was performed using GraphPad Prism software.

For the indirect cAMP assay, changes in cAMP signaling were measured by co-transfection of receptors with a CRE-β-galactosidase (CRE-β-gal) reporter construct (11). Cells were stimulated for 6 h at 37°C with RLN peptides or ML290 at various concentrations. A non-linear regression sigmoidal dose–response curve was then produced using GraphPad Prism. All experiments were conducted at least three times with three to four replicates each time.

### Cell total and surface expression assay of RXFP1

HEK293T cells were transfected with RXFP1 plasmid DNA or empty vector pCR3.1 as described above. After 24 h incubation at 37°C, cells were harvested in PBS/5 mM EDTA. To determine surface expression, 0.5 × 10^6^ cells were fixed in stain buffer (2% BSA/PBS) containing 3.7% formaldehyde, washed, and incubated with 0.5 μg anti-FLAG M1 Ab (Sigma) for 30 min at 4°C. After washing, the cells were then incubated with 1 μg Alexa Fluor 488 goat anti-mouse IgG (Life Technologies) for 20 min at 4°C. Cells were washed and resuspended in stain buffer for analysis on an Accuri C6 flow cytometer (BD Biosciences, San Jose, CA, USA). For total expression, 0.2% Tween-20 (Bio-Rad) was added to the stain buffer at all steps and the cells were processed identically. Cells transfected with empty vector were used as the negative cut-off to determine RXFP1 expression. All experiments were repeated three times in triplicates. Differences in receptor expression were quantified as the ratios of surface expression to total expression, and analyzed with a one-way ANOVA.

### Ligand-binding assays

Saturation-binding studies using Eu-labeled human H2 RLN (Eu-H2 RLN) were performed on whole cells as described previously (12). Cells stably expressing RXFP1 (13) or with a semi-stable transfection of R1-RXFP1 selected using FACS (14) were used in this experiment. Increasing concentrations of Eu-H2 RLN (0.1–50 nM) were utilized and non-specific binding was determined in presence of 1 μM of unlabeled H2 RLN. Readings were taken in triplicate and read on a BMG PolarStar plate reader in clear-bottomed, opaque-walled 96-well plates (PerkinElmer). All experiments were repeated three times. Data were analyzed using GraphPad PRISM and presented as the mean percentage specific binding ± SEM of independent experiments. A non-linear regression one-site binding curve was then fitted and resulting pKd, and Bmax values were subjected to one-way ANOVA and uncorrected Fisher’s LSD comparison test. In this experiment, the cell total and surface expression was analyzed using a previously described method (15). Differences in receptors expression were assessed using a Student’s *t*-test.

## Results

### RXFP1 genes

The ENSEMBL sequence of rhesus macaque RXFP1 (RXFP1-202 ENSMMUT00000041571) had high homology to the human RXFP1 sequence at both the mRNA and amino acid level. The 18-exon structure encoded 757 amino acids with 99% identity to the human protein after removing 22 extra amino acids at the N-terminus (Figure 1). Of the 10 substitutions identified, two were located in the N-terminal signal peptide. The LDLa and LRRs were identical between the two species, as well as ECL3, which is important for the ML290 response. Analysis of the pig RXFP1 annotated sequence revealed the absence of the first exon, but three additional 5′ small exons with no homology to the human or mouse sequence. Using human exon 1 as a probe, we performed a BLAST search of the 50 kb pig genomic sequence upstream of the putative *RXFP1* exon 2. The search revealed exon 1 of pig *RXFP1*, separated from exon 2 by a 42 kb intron, a size comparable to hRXFP1 intron 1. All intron–exon boundaries contained conserved GT and AG sequences at the 5′- and 3′-ends of the introns, which are required for proper RNA splicing. Alignment of the resulting full-length 2277 bp sequence with human cDNA showed 91% identity. The pig RXFP1 758 aa protein sequence was 92% identical to the human sequence (Figure 1A). The second valine in the TM6 domain, adjacent to ECL3, was substituted for leucine in the pig sequence (*V L/V* K F L S L L Q V E I P *G T*). As both macaque and pig cDNAs showed high homology to the human sequence, we chemically synthesized corresponding cDNAs and cloned them into a BacMam vector. Mouse and human cDNA expression BacMam vectors were also produced.

Analysis of the guinea pig annotated RXFP1 genomic sequence and cDNA sequences identified 17 exons with high homology to the corresponding human exons. A BLAST search of the upstream genomic sequence revealed that exon 1 was separated from exon 2 by a 42.8 kb intron. The putative full-length *G-RXFP1* cDNA was 2268 bp long, encoding a 755 aa protein. At the amino acid level, the guinea pig RXFP1 sequence was 84 and 80% identical to the human and mouse RXFP1 proteins, respectively (Figure 1A). The ECL3 sequence was identical to the pig sequence. Since the guinea pig sequence was more divergent from the human and mouse sequences, we used RT-PCR with primers designed from the established first and last exons to generate full-length cDNA. The RT-PCR fragments were obtained from total ovarian guinea pig RNA. Comparison of the sequenced cDNA clones with the genomic sequence from GenBank identified only one synonymous substitution.

**Figure 2:**
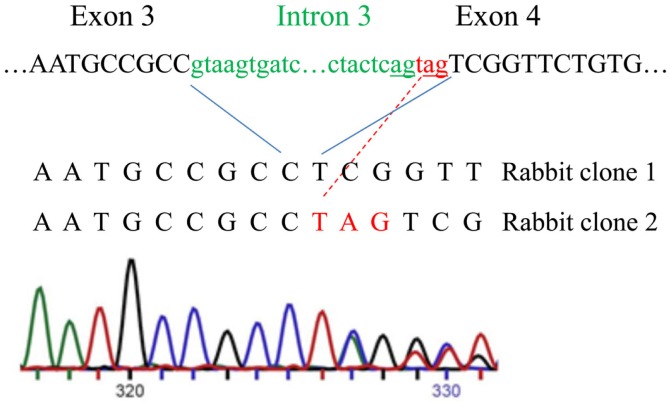
**Alternative splicing of the intron 3 and exon 4 in rabbit RXFP1 genomic DNA**. The upper sequence shows the intron (green) and exon (black) boundaries in the genomic DNA. Three additional nucleotides (in red) are added to the mRNA as result of alternative splicing, as shown with the red line. Below is the sequencing chromatogram showing the presence of two sequences after the alternative splicing site. Note an equal size of the peaks in the overlapping sequence, indicating an equal representation of the two variants in the total mRNA pool.

Next, we analyzed rabbit genomic DNA. Both the cDNA and predicted protein sequence of rabbit RXFP1 were quite different from the human and mouse sequences, with the 3′ exons not well defined. We used RT-PCR with primers designed from the first and the last exons and total uterine rabbit RNA to obtain the expected 2.3 kb full-length sequence of *R-RXFP1*. The fragments were cloned into pCR3.1 vector, and sequencing of the resultant cDNA clones revealed two variants differing by 3 bp in the 5′end (Figure 2). Comparison with the genomic sequence suggested that the variant with the additional TAG sequence was a result of alternative splicing at the 5′-end of exon 4. We performed direct sequencing of the RT-PCR products to confirm the presence of both variants in total mRNA. As shown in Figure 2, the chromatogram depicts a single sequence at the end of exon 3, followed by two overlapping sequences present at equal ratios, as evident by the heights of the overlapping nucleotide picks. Alignment with the genomic sequence showed that the 2277 bp (or 2280 bp) cDNA was encoded by the 18 exons as in other species. When compared to GenBank genomic sequence, all putative exon–intron boundaries, with the exception of the beginning of exon 4, were conserved in rabbit cDNAs. Four synonymous differences were found in our cDNA versus GenBank genomic sequence. At the amino acid level, rabbit RXFP1 sequence was 84 and 79% identical to human and mouse proteins, respectively. The ECL3 sequence was identical to the pig sequence (Figure 1A).

Multiple sequence analysis showed the primate and rodent RXFP1s grouped together, with the pig RXFP1 sequence situated between them (Figure 1B). Rabbit RXFP1 was the most diverged. In rabbit RXFP1, there were multiple substitutions in amino acid positions conserved among the other species, including in the extracellular, 7TM, and C-terminal part (Figure 1A).

### Macaque and pig RXFP1 receptors respond to RLN and ML290 in a CRE-reporter cAMP assay

**Figure 3:**
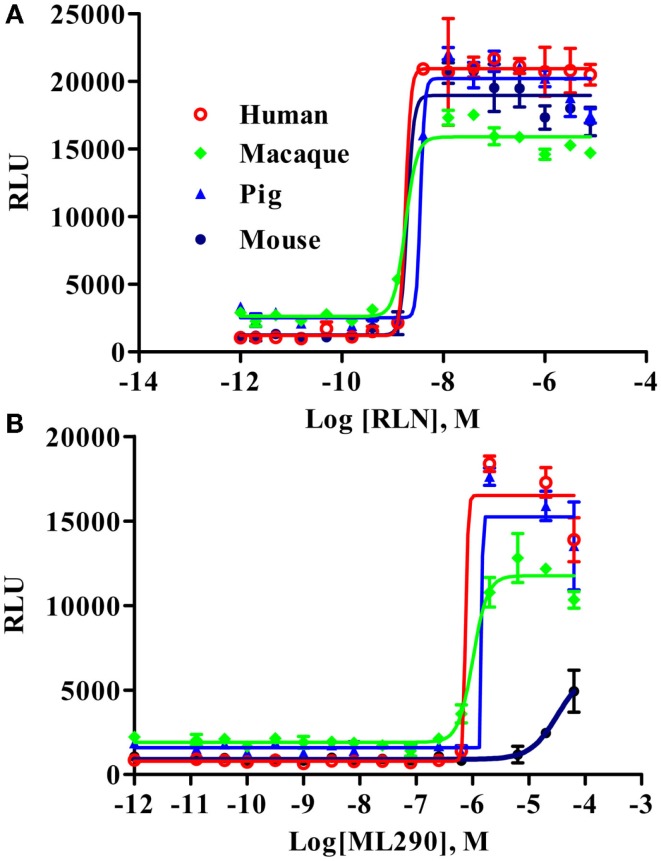
**Activation of macaque and pig RXFP1 receptors by RLN and ML290**. **(A)** Porcine RLN-induced cAMP response. **(B)** ML290-induced cAMP response. HEK293T cells with CRE-luc reporter were transduced with BacMam RXFP1 expression vectors. RLU, relative luciferase units. Data are expressed as mean ± SEM.

To analyze the functional activity of the synthesized macaque and pig RXFP1s, we used a CRE-Luc BacMam luciferase assay (Figure 3). Human and mouse RXFP1s were used in these experiments as controls. The cells were stimulated for 2 h, and the elevation of cAMP production was detected by increased luciferase activity. All four receptors showed similar EC50 when treated with porcine RLN (Figure 3A). Human, macaque, and pig RXFP1s also responded strongly to ML290 stimulation (Figure 3B; Table 1). Previously, we did not see an increase of cAMP in cells transfected with the mouse receptor in a direct cAMP HTRF assay (5). In this experiment, there was significant increase in luciferase activity in cells expressing mouse RXFP1 receptor in response to the highest concentrations of ML290.

**Table 1:** **Activation of RXFP1 receptors with relaxin and ML290**.

Ligand	RLN	ML290
**Receptor**
Human	***	***
Macaque	***	***
Pig	***	***
Guinea pig	***	No
Guinea pig/human	***	*
Rabbit 1 and 2	No	**
Human/rabbit	**	**
Rabbit 1(2)/human	No	**
Mouse	***	No

*Weak, **intermediate, ***strong activation of the receptor.

### Characterization of guinea pig RXFP1

**Figure 4:**
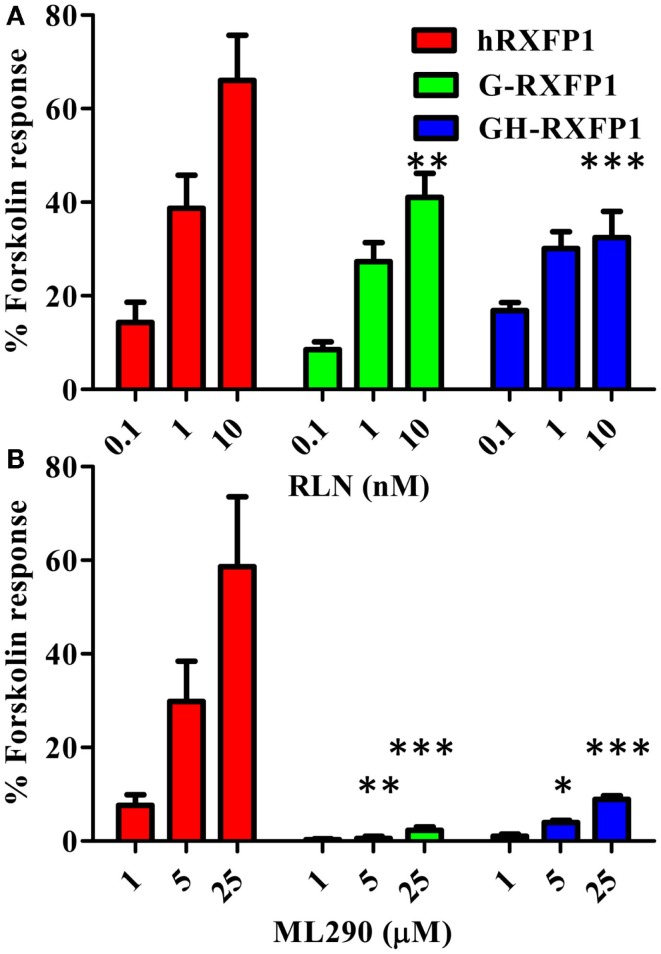
**Activation of guinea pig and guinea pig–human chimeric receptors by RLN and ML290**. **(A)** Porcine RLN-induced cAMP response. **(B)** ML290-induced cAMP response. HEK293T cells were transiently transfected with RXFP1 expression vectors and cAMP was determined using HTRF cAMP assay. cAMP activity is expressed as the percentage of 10 μM Forskolin-stimulated response. Data are expressed as mean ± SEM, each point represent triplicate measurements. The experiment was repeated three times. *p < 0.05, **p < 0.01, ***p < 0.001 compared to hRXFP1.

G-RXFP1 response to RLN and ML290 treatment was tested by measuring cAMP production in HEK293T cells transiently transfected with receptor. A direct HTRF assay to measure cAMP concentration was used in these experiments, and cells transfected with hRXFP1 were used as a control. As shown in Figure 4, G-RXFP1 responded to porcine RLN, although the maximum response was lower than that of hRXFP1. There was small response to ML290 only at highest concentration of compound used. A chimeric guinea pig receptor containing the TM3-C-terminal end of human RXFP1 was created (Figure 1); analysis of cAMP production in cells transfected with the chimeric receptor GH-RXFP1 showed ML290 responsiveness, albeit to a much lower level than in hRXFP1 (*p* < 0.001) (Figure 4; Table 1).

### Characterization of rabbit RXFP1

**Figure 5:**
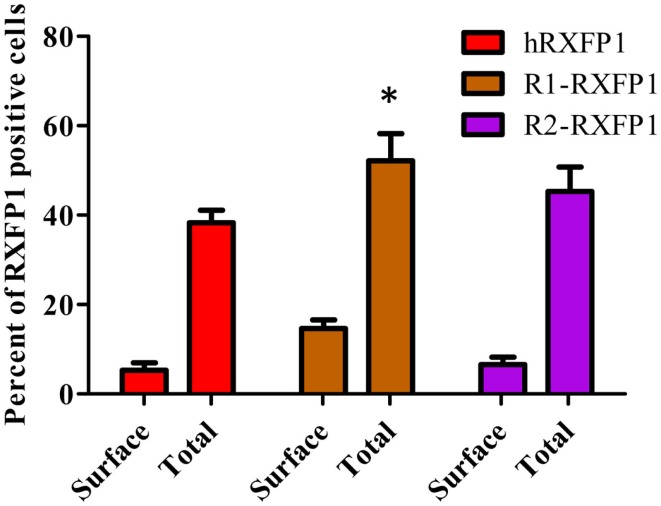
**Expression of two rabbit RXFP1 receptors compared to human RXFP1**. Shown is the total and cell surface RXFP1 expression in transiently transfected HEK293T cells. Data are expressed as mean ± SEM, each point represent triplicate measurements. The experiment was repeated three times. *p < 0.05 compared to hRXFP1.

**Figure 6:**
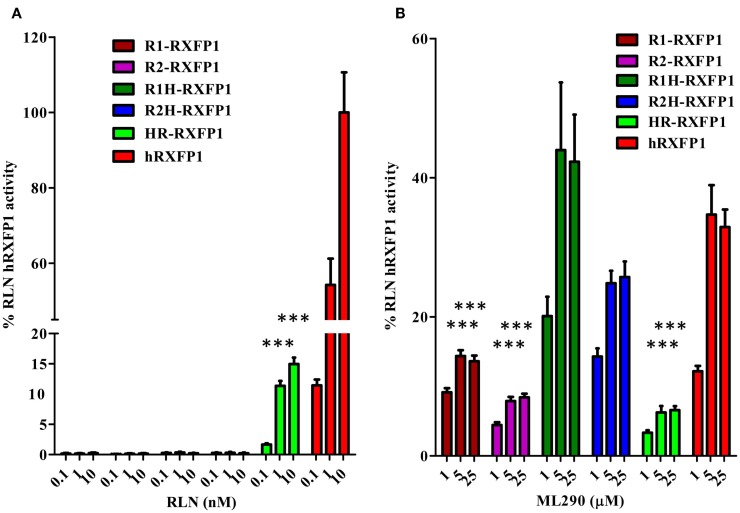
**Activation of rabbit and rabbit–human chimeric receptors by RLN and ML290**. **(A)** Porcine RLN-induced cAMP response. **(B)** ML290-induced cAMP response. HEK293T cells were transiently transfected with RXFP1 expression vectors and cAMP was determined using the HTRF cAMP assay. cAMP activity is expressed as the percentage of hRXFP1 cAMP activation by 10 nM of RLN. Data are expressed as mean ± SEM; each point represents triplicate measurements. The experiment was repeated at least three times. ***p < 0.001 compared to hRXFP1.

Two variants of rabbit *RXFP1* cDNA with and without the additional amino acid in the LDLa-LRRs linker (R1-RXFP1 and R2-RXFP1, respectively) were used in a transient transfection of HEK293T cells, and the cAMP response to ligand treatment was measured. Both failed to respond to porcine RLN, but generated an increase in cAMP after stimulation with ML290 (data not shown). One explanation for the lack of receptor activation could be poor expression of the rabbit receptors on the cell membrane. To test for cell surface expression, we used modified R1- and R2-constructs with a FLAG-tag at the N-terminal part of the receptor. Both receptors were expressed at the same or greater levels relative to a FLAG-tagged human RXFP1 (Figure 5). The HTRF assay on FLAG-tagged rabbit receptors failed to detect cAMP production when they were stimulated by porcine RLN. Stimulation with ML290 produced a cAMP increase in cells transfected with both rabbit receptors, albeit with lesser efficacy than hRXFP1 (Figure 6).

Two chimeric constructs were created to identify the region of the rabbit receptor responsible for its lack of RLN response: R1H-RXFP1 and R2H-RXFP1, which contain most of the ectodomain of the two rabbit receptors and the 7TM domain of hRXFP1; and HR-RXFP1, which contains most of the hRXFP1 ectodomain and rabbit 7TM (Figure 1). The N-terminus contains LRR4, 5, 6, and 8, which have been identified as the sites of RLN binding (2). As shown in Figure 6A, R1H-and R2H-RXFP1 were inactive when stimulated with porcine RLN, whereas HR-RXFP1 responded at a low level to RLN stimulation. While all three receptors respond to ML290, the level of activation of R1H- and R2H-RXFP1 was higher than that of R1-, R2-, or HR-RXFP1-transfected cells (Figure 6B; Table 1).

**Figure 7:**
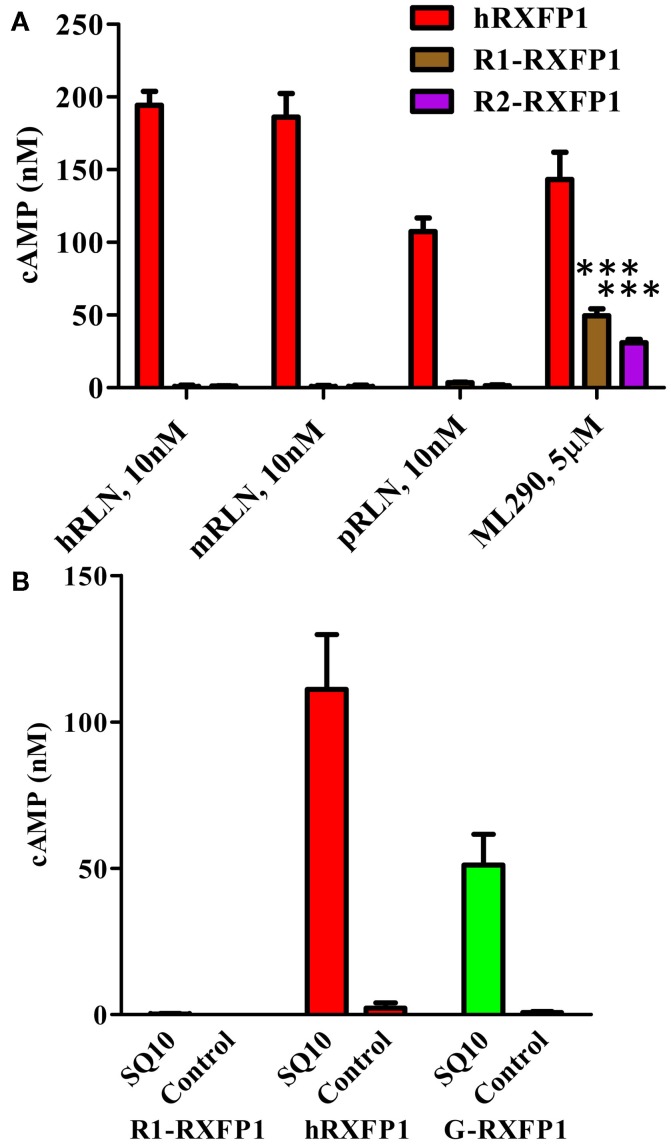
**Activation of rabbit receptors by relaxin peptides from different species**. **(A)** Activation of two rabbit receptor variants (R1- and R2-RXFP1) and hRXFP1with 10 nM of human (hRLN), mouse (mRLN), and porcine (pRLN) relaxin peptides, and by 5 μM of ML290. Treatments of rabbit RXFP1 are statistically significant compared to hRXFP1, ***p < 0.001. **(B)** Activation of rabbit (R1-), human (h), and guinea pig (G-) RXFP1s with rabbit relaxin SQ10. Conditioned media from HEK293T cells transfected with SQ10 and control empty vector was used for cell stimulation. cAMP activity is expressed as the percentage of 10 μM Forskolin stimulation for each type of cells. Data are expressed as mean ± SEM; each point represents triplicate measurements. The experiment was repeated at least three times. Treatment of R1-RXFP1 is statistically significant (*p* < 0.0001) compared to hRXFP1 or G-RXFP1.

In addition to porcine RLN, we analyzed the response of the two rabbit RXFP1 variants to human and mouse RLN in the HTRF cAMP assay (Figure 7A). In all cases, 10 nM of peptide failed to stimulate cAMP production in cells transiently transfected with rabbit receptors, whereas they responded to 5 μM of ML290. Human receptor was active with all ligands.

To test the activity of rabbit receptor versus rabbit RLN (SQ10), we designed an expression construct of the latter gene. HEK293T cells were transfected with SQ10, and conditioned medium was used for activation of cells expressing rabbit, human, and guinea pig RXFP1s. Cells transfected with the latter two receptors responded to SQ10 treatment with cAMP production measured by the HTRF assay (Figure 7B). No response was recorded from cells transfected with R1- or R2-RXFP1, suggesting that rabbit RLN does not induce cAMP production through RXFP1 in rabbits.

**Figure 8:**
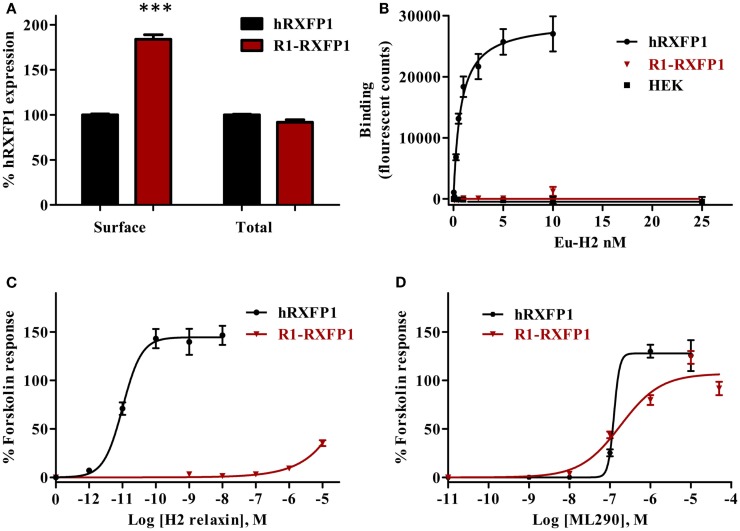
**Rabbit RXFP1 does not bind relaxin peptide**. **(A)** Total and cell surface expression of hRXFP1 and R1-RXFP1. Expression is normalized to the expression of human receptor. ***p < 0.001 compared to hRXFP1. **(B)** Saturation binding using Eu-labeled H2 RLN. **(C)** Human RLN-induced cAMP response. **(D)** ML290-induced cAMP response. cAMP activity is expressed as the percentage of the 5 μM Forskolin-stimulated response for each receptor. Data are expressed as mean ± SEM; each point represents triplicate measurements. The experiment was repeated at least three times.

Binding of Eu-human RLN to the rabbit R1-RXFP1 receptor was evaluated in a saturation binding assay using selected HEK293T cells with a high level of RXFP1 (Figure 8). The R1-RXFP1 was well-expressed on the cell surface membrane, with an even somewhat higher level than hRXFP1 (Figure 8A), similar to what was detected in transiently transfected cells. These cells were used in human RLN-binding experiments. While strong binding of labeled RLN was detected with human RXFP1, no binding to rabbit R1-RXFP1 receptor was found (Figure 8B). In the CRE-reporter-based cAMP assay, hRXFP1 and R1-RXFP1 were stimulated with human RLN and ML290. For ML290 treatment, the cAMP response in cells transfected with rabbit receptor measured using this approach was comparable to hRXFP1. At very high concentrations of RLN (>1 μM), there was some increase in β-gal activity for R1-RXFP1-transfected cells (Figures 8C,D).

## Discussion

Due to its vasodilatory, antifibrotic, and cytoprotective properties, among others, RLN hormone induces pleotropic responses in multiple normal and diseased tissues and organs (2). In animal models, treatment with RLN has shown therapeutic effects in induced liver, pulmonary, kidney, and heart fibrosis, as well as diabetes, ischemia–perfusion injury, wound healing, and other common diseases. Most of these studies were performed with rodents, although other species were used to a lesser degree. However, there are noted differences in the physiology of this hormone in other species; for instance, the concentration of RLN during pregnancy in mice is 100 times higher than in women (1). The largest clinical trial of the therapeutic use of RLN was in acute heart failure patients (4). The data indicated the possible benefits of even short-term intravenous RLN administration. We have recently reported the discovery of the first small-molecule agonist series for the RLN receptor (5, 6). This has raised the possibility of developing a therapeutic agent with high stability, activity, specificity, and potentially oral delivery. However, we have shown that the identified compounds did not activate the mouse RLN receptor. Using chimeric human–mouse receptors and site-specific mutagenesis, we have determined that the amino acid sequence of the ECL3 of the 7TM determines the receptor selectivity. The mutation of four amino acids in the mouse receptor ECL3 made it responsive to small agonist treatment. Analysis of the available sequences in GenBank revealed that both rat and hamster RXFP1s have the mouse variant of ECL3, and thus these rodents could not be used for the analysis of small molecules. Here, we have cloned and tested the functional activation of RXFP1s from mammals of four different orders, Primates (rhesus macaque), Artodactyla (pig), Lagomorpha (rabbit), and Rodentia (guinea pig). The data show that the RXFP1 receptors from the first two species are fully activated by RLN and ML290 and hence, the corresponding animal models can be used for RLN and ML290 studies. Guinea pig RXFP1 does not respond to ML290. The use of rabbit in studies of RLN biology should be further critically assessed as in our experiments RLN did not bind to the rabbit RXFP1 receptors nor did it activate cAMP response at physiologically relevant concentrations (Table 1).

The availability of genome sequencing data allows quick retrieval of gene information. However, a critical appraisal of this information is required. In the case of RXFP1, the genomic structure of cloned human, mouse, and rat receptors is well-established and each comprises 18 coding exons (16). The same conserved structure was annotated for macaque and pig genes. In the case of guinea pig and rabbit, we identified the missing first exon using a BLAST search of the genomic DNA. The correct assembly of the ORFs was further confirmed by RT-PCR isolation of the full-length cDNAs, cloning, and sequencing. We found two splice variants of the rabbit receptor mRNA, an apparent consequence of a mutation in the splice site of the intron 3/exon 4 boundary, which results in one extra amino acid in the LDL–LRR linker. While splice variants of hRXFP1 affecting full exons were described (13, 17, 18), such additional insertion of only three nucleotides is unique for rabbit RXFP1.

Comparison of the various sequences indicated that pig, rabbit, and guinea pig receptors share the same ECL3 sequence, differing from the human and monkey sequence by only one amino acid (V647). Multiple substitutions were found in the rabbit sequence, including three unique substitutions in the LDLa domain, numerous changes in the LDLa–LRR linker (17 unique amino acids) and in several LRR repeats. Notably, all previously identified amino acids essential for the structural and functional integrity of human RXFP1 remained conserved in the rabbit receptor: amino acids required for the coordination of Ca^2+^ binding and receptor activation in the LDLa domain (19, 20), previously described LRR amino acids important for primary RLN binding by the RXFP1 ectodomain (2), and the sites crucial for secondary binding of RLN peptide to the ECL1 or ECL2 (21). However, there is a substitution of serine at amino acid 33, which is proline in all the other RXFP1 receptors. This residue is located next to one of the crucial cysteine residues in the LDLa module that are essential for its function. It is possible that this substitution results in a structural perturbation in the LDLa module. Additionally, there are numerous proline substitutions in the LDLa–LRR linker region which would result in structural changes in this domain. Recent studies have suggested that this linker region may have an important role in receptor activation by the LDLa module (14).

Analysis of RLN response measured by cAMP production revealed RLN activity in all but the rabbit receptor. Neither of the two rabbit RXFP1 variants responded to stimulation with RLN peptides from various mammalian species, including previously described rabbit RLN homolog SQ10 (22). Moreover, we were not able to detect binding of labeled human recombinant RLN to the rabbit receptor. Taken together, the homology of the isolated rabbit clones with other species RXFP1s, almost identical sequence of our cDNAs to the GenBank genomic DNA, and identity of the cloned cDNA with the DNA obtained by direct sequencing of the isolated RT-PCR fragments all suggest that we have isolated the correct full-length rabbit RXFP1 clones. The rabbit receptors were activated by ML290, indicating their functionality.

It was recently suggested that there are five copies of the RLN gene in the rabbit genome, which raises the question of whether the rabbit RLN peptide used in our experiments is correct (23). Putative translation of two rabbit RLN genes produced identical peptides. The three other RLN genes encoding putative rabbit RLN peptides each differ from the first two by a single evolutionary non-conserved amino acid (23). The sequence of the first peptide was also identical to the previously reported sequence encoded by SQ10 cDNA obtained by RT-PCR (9) and partial protein sequencing (24). Therefore, we decided to use SQ10 cDNA in our experiments to generate rabbit RLN. Importantly, it activated human and guinea pig receptors and thus was fully functional. Taking into account that there is only a single amino acid difference between the SQ10 sequence and other three putative peptides, it seems highly unlikely that the latter RLNs will activate rabbit RXFP1.

Surprisingly, in an indirect assay, activation of a CRE-reporter was detected when RLN was used at a concentration far exceeding the detected serum RLN range in rabbits (25). Similarly, activation of the CRE-reporter was observed with high-dose ML290 treatment on the mouse receptor, despite no activation being seen in the direct HTRF cAMP assay. One explanation is that ligand interactions with the receptor triggered signaling pathways other than cAMP in both CRE-reporter assays, which then affected CRE transcriptional activity (26). It was demonstrated that RLN activated various signaling pathways in cells expressing RXFP1 (27, 28). It should be noted, however, that the fact that we were not able to detect human RLN binding to the rabbit receptor in our assays contradicts this suggestion.

Another possible explanation for the loss of activity of rabbit receptors is that the activation of rabbit receptor by RLN requires dimerization or interaction with other GPCRs or other cellular partners. It is possible that such partners might be rabbit-specific or that the receptor works only in rabbit cells. Such interactions have been shown; for instance, RXFP1 can directly interact with the angiotensin II type 2 receptor to regulate downstream cellular signaling (29). It is also possible that rabbit RLNs do not signal through RXFP1, and other ligands activate this receptor. In any of these scenarios, the question of whether rabbit is an appropriate model for RLN studies should be carefully examined. One might wonder if treatment of rabbits rather than guinea pigs with serum from the pregnant animals in Dr. Hisaw’s original experiments would have led to the discovery of RLN.

The rabbit and guinea pig receptors provide new structural templates for analysis of RLN and ML290 activation of RXFP1. Using chimeric human and rabbit receptors, we showed here that the extracellular part of the rabbit receptor is responsible for the failure of RLN activation. Indeed, it was shown that primary binding of RLN to RXFP1 involve sites within LRR4, 5, 6, and 8 (2). In contrast, the 7TM region of RXFP1, which is the site of allosteric small-molecule agonist binding, is functional in rabbits. The opposite was true for guinea pig RXFP1: despite having the same ECL3 sequence as pig or rabbit RXFP1, the receptor was activated by ML290 only at highest concentration of ML290 and with much lower efficacy than hRXFP1. As noted above, one potential site of ML290 interaction with the receptor is ECL3 (5). To further define the region of interaction, we substituted the C-terminus guinea pig fragment with human sequence in chimeric GH-RXFP1. This part contained peptide regions adjacent to ECL3: TM3–7, ICL2–3, and ECL2–3. The data showed that the efficacy of ML290 stimulation was improved, however, it was still lower than in hRXFP1-transfected cells. Thus, specific amino acids in the guinea pig TM1–3, or their interaction with TM3–7 amino acids might be responsible for ML290 binding or activation. Importantly, a modified mouse receptor with humanized ECL3 is activated by ML290 (5). Thus, amino acid substitutions unique for the guinea pig 7TM domain and not present in other species might be responsible for the lack of activation with ML290. Identification of such sites might help in understanding the structural basis of ML290 and RXFP1 interactions. Collectively, our data demonstrate that different parts of the receptor are important for RLN- or ML290-induced activation and thus indicate the allosteric mode of activation by two ligands.

In summary, the information derived from this study may help in the selection of appropriate animal models to study the biological effects of RLN and ML290. The comparisons of RXFP1 sequences have provided further insights into the structural basis, mechanism of activation, and selectivity of peptide and small-molecule agonists for RXFP1.

## Conflict of Interest Statement

The authors declare that the research was conducted in the absence of any commercial or financial relationships that could be construed as a potential conflict of interest.

## Footnotes

^1^http://www.ensembl.org

^2^www.ncbi.nlm.nih.gov

^3^http://www.ebi.ac.uk/Tools/msa/
